# Comprehensive Analysis of Somatic Reversion Mutations in Homologous Recombination Repair (HRR) Genes in A Large Cohort of Chinese Pan-cancer Patients

**DOI:** 10.7150/jca.65650

**Published:** 2022-01-09

**Authors:** Hong Zong, Jian Zhang, Zhengyang Xu, Jia-Ni Pan, Rong Wang, Jinming Han, Miao Jiang, Ruiping Ren, Li Zang, Haitao Wang, Wen-Ming Cao

**Affiliations:** 1 Department of Oncology, The First Affiliated Hospital of Zhengzhou University, Zhengzhou, China.; 2 Department of Medical Oncology, Fudan University Shanghai Cancer Center, Shanghai, China.; 3 Department of Oncology, Shanghai Medical College, Fudan University, Shanghai, China.; 4 Department of Tumor Radiotherapy and Chemotherapy, The Affiliated People's Hospital of Ningbo University, Ningbo, China.; 5 Department of Breast Medical Oncology, Cancer Hospital of the University of Chinese Academy of Sciences (Zhejiang Cancer Hospital), Hangzhou, China.; 6 Institute of Cancer and Basic Medicine (ICBM), Chinese Academy of Sciences, Hangzhou, China.; 7 Zhejiang Chinese Medical University, Hangzhou, China.; 8 Department of Oncology, The Second Hospital of Tianjin Medical University, Tianjin, China.

**Keywords:** Homologous recombination repair, Reversion mutation, Next-generation sequencing, Platinum-based chemotherapy, PARP inhibitor

## Abstract

**Purpose:** Mutations leading to homologous recombination deficiency (HRD) increase the tumor sensitivity to platinum-based chemotherapy and PARP inhibitors. However, reversion mutations often develop conferring acquired drug resistance. There is still a lack of comprehensive investigation on HRR reversion mutations in large pan-cancer cohorts, especially in the Eastern Asian population. This study aims to characterize reversion mutations in homologous recombination repair (HRR)-related genes in a large cohort of Chinese pan-cancer patients.

**Methods:** Sequencing data from 23,375 patients across over 17 cancer types were retrospectively analyzed for pathogenic/likely pathogenic (P/LP) germline mutations in 15 HRR genes. Somatic mutations detected in tumor or circulating cell-free DNA predicted to restore the open reading frame of the deleterious allele were subsequently identified as reversion mutations.

**Results:** 654 cases out of 23,375 (2.8%) unselected pan-cancer patients were identified with HRR germline mutations. Secondary somatic mutations were further analyzed in their matched tumor/plasma samples. The overall frequency of reversion mutation was 1.7% (11/654). The reversion mutations occurred only in 3 out of the 15 HRR genes: *BRCA1* (3.8%), *BRCA2* (3.5%) and *PALB2* (2.0%) from 11 patients (6 breast cancers, 1 ovarian cancer, 1 pancreatic cancer, 1 lung cancer and 2 breast and ovarian dual cancers). We identified total 25 reversion events (*BRCA1*, n=9; *BRCA2*, n=8; *PALB*2, n=8), including 12 pure deletions, 10 missense single nucleotide variants, 2 insertions and 1 splice site mutation. Besides, we detected microhomology length >1bp in seven out of the reversion deletions (58.3%), suggestive of microhomology-mediated end-joining (MMEJ) repair signature. Intriguingly, a positive correlation (r=0.85, p=0.001) between the length of deletion and the microhomology length was also observed. We obtained disease courses from 6/11 patients with reversion events. Four acquired reversions after the failure of the PARP inhibitor treatment. Two patients had somatic reversion mutations identified after progressing on platinum-based treatment.

**Conclusion:** This study comprehensively depicts the prevalence and characteristics of HRR reversion mutation of germline mutations in an unselected Chinese pan-cancer cohort. The reversion mutations predominantly occurred in *BRCA1*, *BRCA2* and *PALB2*. The results revealed that reversion mutations frequently occurred after resistance to platinum-based chemotherapy and/or PARP inhibitor. Our study provides insight into the underlying mechanism of drug resistance in HRD tumors and suggests that monitoring HRR mutation status along the disease course could be beneficial especially for informing resistance mechanisms and guiding subsequent therapies.

## Introduction

Detecting and repairing DNA damage is pivotal for maintaining normal cell function and genomic stability. Homologous recombination repair (HRR) system, using the sister chromatid or homologous chromosome as the template to restore DNA, is the most fidelity mechanism in repairing double-stranded DNA breaks (DSBs) 1. The HRR-related mutations are predominantly present in the Fanconi anemia (FA)-BRCA pathway 2, 3. Those mutations, mainly frameshift, nonsense, splice site, and nonstart mutations, could lead to truncated protein translation. Dysfunction of the HRR mechanism confers the genomic instability that ultimately leads to carcinogenesis.

Germline mutations in HRR genes *BRCA1/2* and *PALB2* are known to associate with hereditary breast cancer and ovarian cancer, which have also been described in pancreatic cancer and prostate cancer. Due to synthetic lethality, patients harboring such mutations are more sensitive to DNA-damaging agents, such as platinum-based chemotherapy and poly ADP-ribose polymerase (PARP) inhibitors 4, 5. With the broader application of platinum-based chemotherapy and the newly approved PARP inhibitors, acquired resistance cases have being ever-increasingly reported 6-8. The HRR function recovery via acquiring secondary mutation is one of the major underlying mechanisms that mediate the resistance 5. Usually the observed secondary somatic mutations are close to the primary mutations, restore the open-reading frame (ORF) of the functional protein, and assist the cell in recovering HRR function 4, 9, 10.

Reversion mutations within multiple genes in the HRR pathway, including *BRCA1*, *BRCA2*, *RAD51C*, *RAD51D*, and *PALB2*, have been reported in ovarian, prostate, and breast carcinomas as a mechanism of acquired or primary resistance to platinum-based chemotherapy and PARP inhibitors 7-18. However, most studies either reported sporadic cases or recruited small cohorts mainly from Western populations. There is still a lack of comprehensive investigation on HRR reversion mutations in large pan-cancer cohorts, especially in the Eastern Asian population.

Here, we characterized the prevalence and spectrum of the reversion mutations in HRR genes across more than 17 different cancer types in a Chinese cohort of 23,375 patients. We also obtained treatment information and clinical outcomes from six cases with reversions to explore the heterogeneity in clinical response.

## Methods

### Patients and study design

We retrospectively recruited patients with solid tumors according to the following criteria: 1) underwent somatic genomic profiling from January 2019 to May 2020 in the Burning Rock LAVA Database; 2) also provided matched white blood cell (WBC) samples for sequencing in parallel for germline mutation filtration; 3) sequenced with targeted panel including 15 HRR-associated genes (*BRCA1, BRCA2, ATM, PALB2, CHEK2, BRIP1, RAD51B, RAD51D, RAD54L, RAD51C, BARD1, FANCI, FANCL, CHEK1, CDK12*). Pathogenic/likely pathogenic (P/LP) germline mutations in 15 HRR-associated genes were screened based on their WBC sequencing results. Frequencies of each mutation were determined for the total cohort, as well as for each cancer type (lung cancer, colorectal cancer, breast cancer, stomach/esophagus cancer, ovarian cancer, pancreatic cancer, sarcoma, liver cancer, cervical cancer, cancer of the biliary tract, endometrial adenocarcinoma, prostate cancer, kidney cancer, head-neck cancer, bladder cancer, malignant melanoma, glioma, and other cancer types). The study was approved by the Ethics Committee of Zhejiang Cancer Hospital. Patient's informed consent was waived due to the retrospective nature of the study. Procedures involving human participants in this study were complied with the Declaration of Helsinki.

### Reversion detection in tumor and circulating cell-free DNA (cfDNA)

For patients identified with P/LP germline HRR mutations, secondary somatic mutations were further analyzed in their WBC matched tumor/plasma samples and their subsequently sequenced samples if applicable. The detected secondary mutations were filtered for potential reversion mutations and further divided into two sub-groups: confirmed and putative. A reversion mutation was classified as “confirmed” if i) it was present on the same sequencing read with the primary germline mutation and predicted to restore the ORF; or ii) both germline and somatic mutations occurred in or shared the same nucleobase(s). Other mutations, which were present in the same gene yet located far away from the germline mutations, were classified as “putative” ones.

### Next-generation sequencing (NGS)

NGS were performed in Burning Rock Biotech, a College of American Pathologists (CAP)-accredited and Clinical Laboratory Improvement Amendments (CLIA)-certified clinical laboratory, as described previously 19, 20. In general, the overall procedure included DNA extraction, library construction, sequencing and data analysis. The cfDNA from plasma or genomic DNA from tumor samples were extracted using QIAamp Circulating Nucleic Acid Kit (Qiagen, Hilden, Germany) following the manufacturing protocol. The DNA libraries were prepared with targeted enrichment by using one of the commercially available panels including 168 genes (Lung Plasma, Burning Rock Biotech, Guangzhou, China), 295 genes (OncoScreen, Burning Rock Biotech, Guangzhou, China), or 520 genes (OncoScreen Plus, Burning Rock Biotech, Guangzhou, China). All panels included the 15 HHR genes. Indexed libraries were subsequently sequenced on Illumina NextSeq 500 system (Illumina, Inc., Hayward, CA, USA) with paired-end reads.

### Sequencing data analysis

The paired-end reads were mapped to the reference genome with Burrows-Wheeler aligner v0.7.10 21. The Genome Analysis Toolkit (GATK) v.3.2 22 and VarScan v.2.4.3 23 were employed for local realignment, variant calling and annotation. Variants with population frequencies of over 0.1% in the Exome Aggregation Consortium (ExAC), 1000 Genomes, dbSNP and ESP6500SI-V2 databases were grouped as single nucleotide polymorphisms (SNPs). ANNOVAR 24 (2016-02-01 release) and SnpEff v3.6 25 were used for the annotation of the remaining variants. All germline variants were manually annotated and categorized into five classes following the American College of Medical Genetics and Genomics and the Association for Molecular Pathology (ACMG/AMP) recommendations as follows: pathogenic (Class 5), likely pathogenic (Class 4), variants of uncertain significance (Class 3,) likely benign (Class 2) and benign (Class 1) 26. All variants classified as P/LP after manual curation were considered for further analysis, while variants classified as of unknown significance, likely benign or benign were disregarded.

### Analysis of microhomologies

The presence of microhomologies in deletions was analyzed as previously described 27, 28. Briefly, if the nucleotides before a pure deletion were identical with the last nucleotides of the deleted sequence, the position of the identical nucleotides was adjusted. The number of contiguous nucleotides at the beginning of the deleted sequence that matched the sequence 3'-flanking the deletion was determined as the length of microhomology. Microhomologies of > 1 bp were defined to possess the signature of microhomology-mediated end-joining (MMEJ) repair.

### Statistical analysis

Differences in proportion between groups were calculated using Fisher's exact test. The correlation between length of deletion and the microhomology length was analyzed by Pearson correlation. P-values <0.05 were considered statistically significant.

## Results

### Prevalence of germline mutations in HRR genes

We retrospectively evaluated the WBC samples of 23,375 solid tumors patients who underwent NGS. The overall cohort consisted of more than 17 cancer types, including lung cancer (n=17,029), colorectal cancer (n=1,282), breast cancer (n=1,024), stomach/esophagus cancer (n=732), ovarian cancer (n=656), pancreatic cancer (n=269; Table 1). Of the total population, the majority (82.5%) were late stage cancer patients (Table S1).

A total of 663 P/LP germline mutations spanning 15 HRR genes were identified in 654 out of 23,375 (2.8%) pan-cancer patients, among which nine patients (2 with breast cancer, 2 with lung cancer, 2 with colorectal cancer and 3 with other cancer types) harbored dual germline mutations and the remaining all carried a single germline mutation. Across all investigated cancer types, ovarian cancer (14.2%, n=93) showed the highest prevalence of HRR germline mutations, followed by cancers of breast (9.8%, n=100), endometrium (6.5%, n=11), and pancreas (5.9%, n=16) (Table 1). No bladder cancer was detected with HRR P/LP germline mutation in this cohort.

Among the 15 HRR genes analyzed, germline mutations occurred predominantly in *BRCA* genes: 175 *BRCA2* and 130 *BRCA1* mutations were observed, comprising 26.4% (175/663) and 19.6% (130/663) of all HRR germline events, respectively. Other commonly mutated genes included *ATM* (n=67, 10.1%), *RAD51D* (n=58, 8.7%), *PALB2* (n=49, 7.4%), *CHEK2* (n=46, 6.9%), *BRIP1* (n=35, 5.3%), and *RAD54L* (n=30, 4.5%) (Figure S1).

### Comprehensive characterization of HRR reversion mutations

Among the 654 patients with germline mutations, 11 cases (1.7%) were identified with reversion mutations including six cases with breast cancer, one with ovarian cancer, one with pancreatic cancer, one with lung cancer and two with breast and ovarian dual primary cancers (Table 2, 3). Breast cancer exhibited the highest prevalence of reversion mutations (8.1%), followed by pancreatic (6.2%) and ovarian cancer (3.2%), while reversion events only occurred in 0.3% of lung cancers (Table 2). The reversion mutations occurred in 3 genes: *BRCA1* (n=5), *BRCA2* (n=5) and *PALB2* (n=1), but not in any other HRR genes,* ATM, CHEK2, BRIP1, RAD51B, RAD51D, RAD54L, RAD51C, BARD1, FANCI, FANCL, CHEK1, and CDK12,* in our cohort. Among the three HRR genes with reversions, *BRCA1* revealed the highest frequency of 3.8%, while 3.5% and 2.0% of *BRCA2* and *PALB2* germline mutations reverted, respectively (Table 2). *PALB2* reversion mutation was only detected from one case with breast cancer.

**Figure 1:**
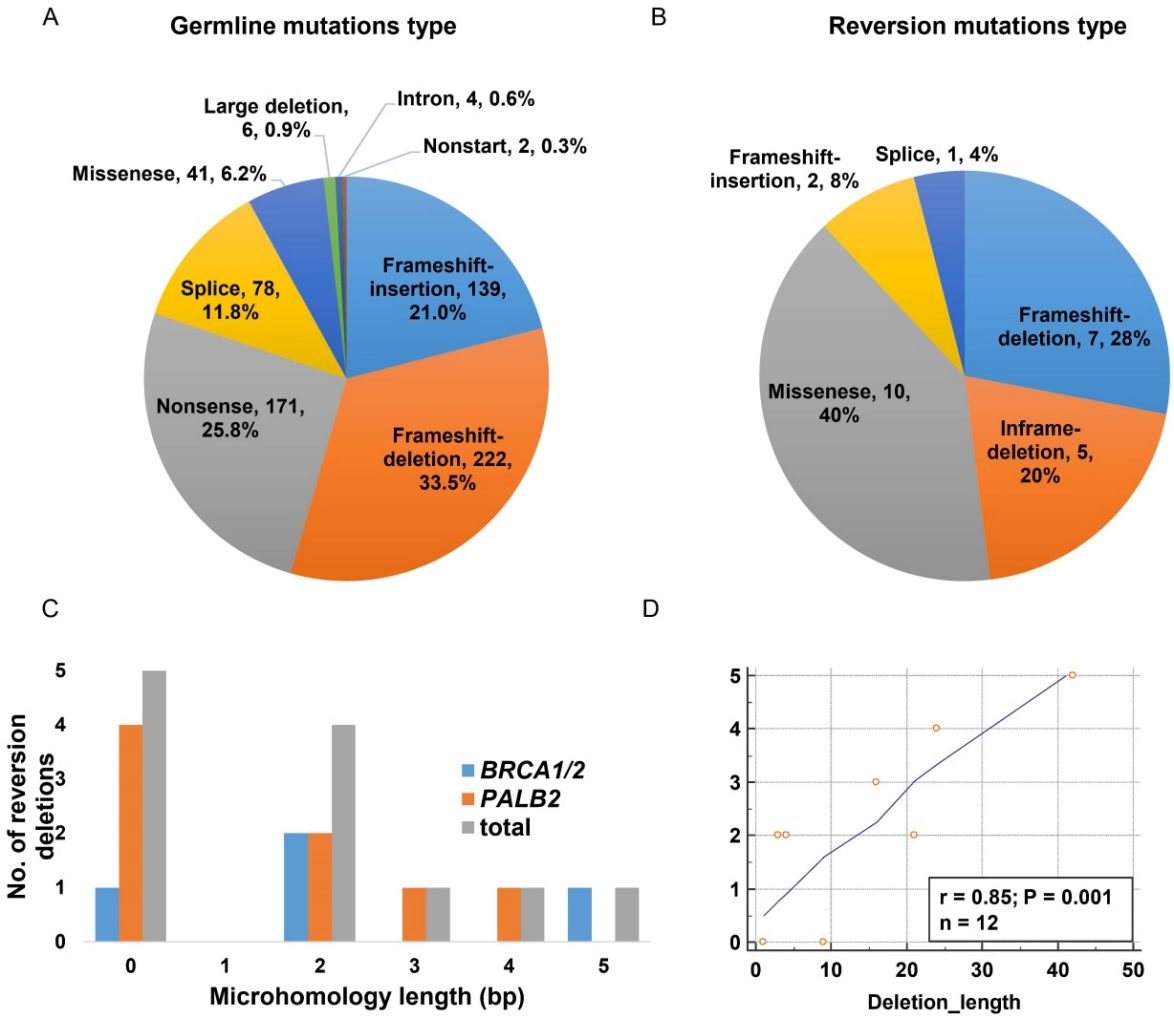
Characterizing of reversion mutations. A. Variant type distribution of the overall germline mutation (n=663); B. Distribution of variant type in somatic reversion mutations (n=25); C. Distribution of microhomology length in somatic reversion deletions; D. The correlation of reversion deletion length and microhomology length.

We further explored the propensity of HRR germline mutations to acquire reversion based on the mutation type. We observed that frameshift (insertion and deletion) and nonsense variants comprised of the vast majority of the overall P/LP germline mutations (54.5% and 25.8%, respectively), while missense and splice-site variants as well as large deletion, intron and nonstart mutations were observed with low frequencies (Figure 1A).

The majority of the reversion mutations were pure deletions causing frameshift and in-frame mutations (48%, n=12, Figure 1B), ranging from 1bp to 42bp and mainly arising in *PALB2* gene (n=8, Table 3). The missense single nucleotide variant (SNV) accounted for 40% (n=10) of reversion events. We further analyzed microhomologies surrounding the 12 reversion deletions and detected microhomology length >1bp in seven of them (58.3%), suggestive of MMEJ repair signature (Figure 1C). The remaining five deletion evens revealed no microhomology features. Intriguingly, we also observed a positive correlation (r=0.85, p=0.001) between the length of deletion and the microhomology length (Figure 1D).

**Figure 2:**
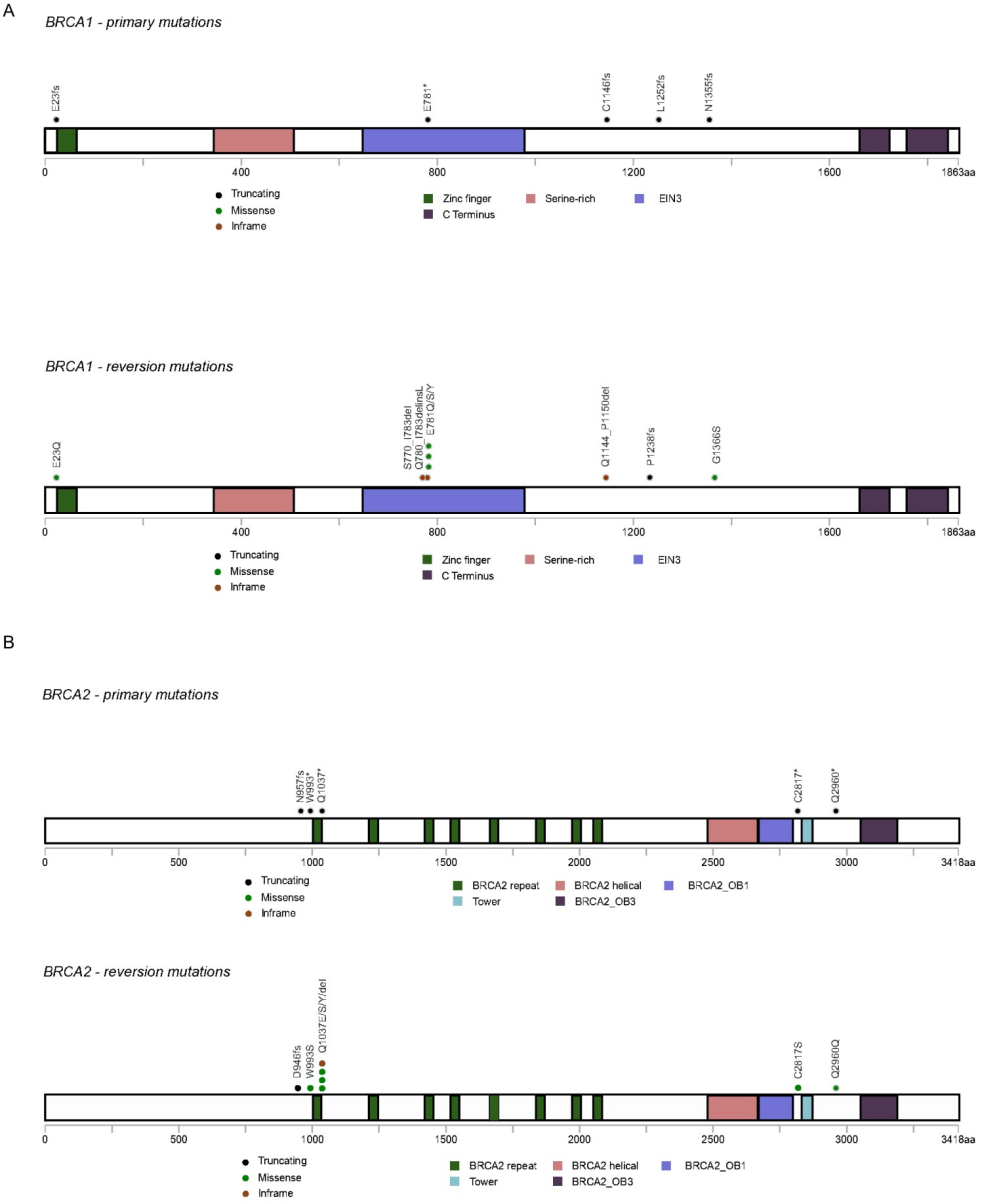
Distribution of reverted primary germline mutations and reversion mutations on *BRCA1* and *BRCA2*. A. *BRCA1*; B. *BRCA2.*

A total of 25 reversion mutations (21 confirmed and 4 putative) were found: nine within *BRCA1*, eight within *BRCA2*, and eight within *PALB2*. Eight out of the eleven patients acquired a single reversion mutation towards the primary germline mutation (Table 3); while Patient 1, 3 and 10 acquired multiple reversion mutations in the context of primary mutation *BRCA1* p.E781*, *PALB2* p.T827fs and *BRCA2* p.Q1037*, respectively. For *BRCA1* gene, reverted germline mutations were mainly located in the hotspot mutated regions in the sequence comprising exon 10 (n=4) and one was observed in exon 2 encoding the Zinc finger ring region (Figure 2A). For *BRCA2* gene, reversion events were found within the region encoding *BRCA2* repeats in exon 11 (n=3), exon 19 (n=1) and exon 22 (n=2) (Figure 2B). *PALB2* reversion mutations were only observed in a single case located in exon 5.

### HRR reversion mutations associated with resistance to platinum-based chemotherapy or PARP Inhibitor

**Figure 3:**
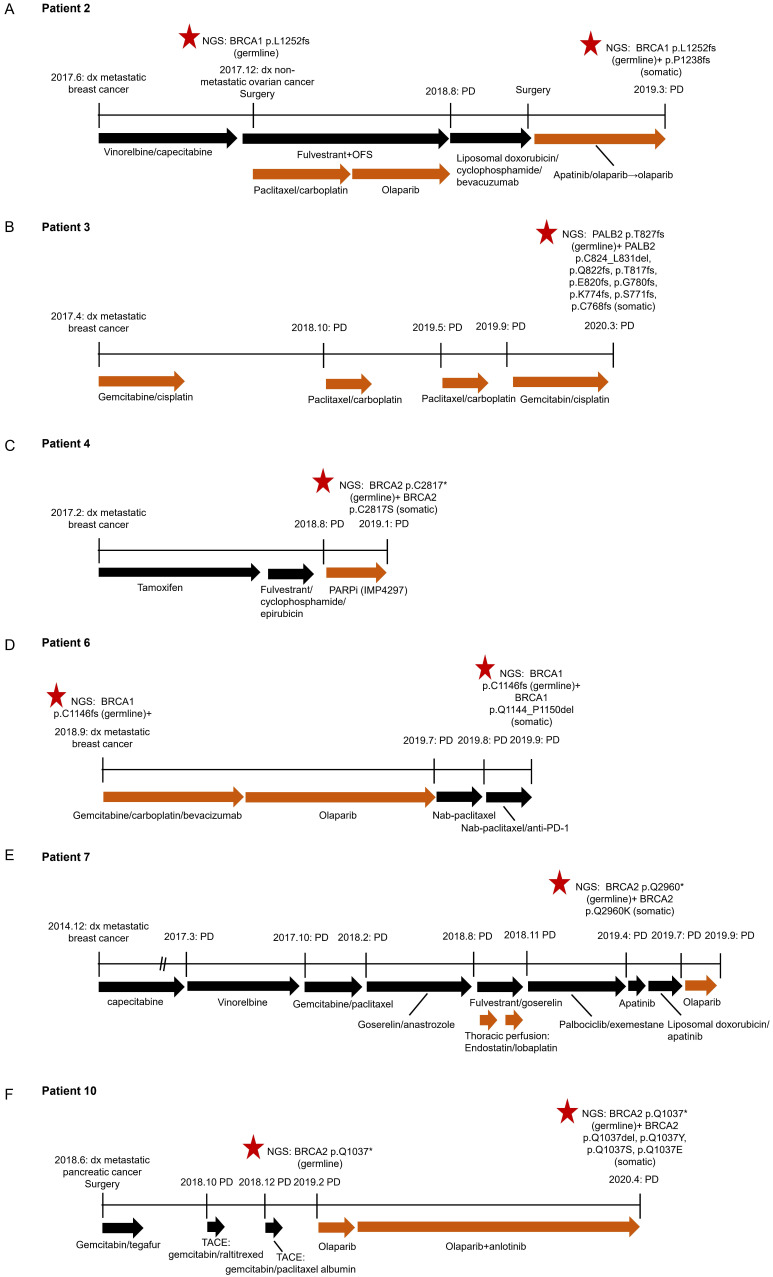
Clinical courses of six patients with reversion mutations. A. Patient 2; B. Patient 3; C. Patient 4; D. Patient 6; E. Patient 7; F. Patient 10. dx, diagnosis. OFS, ovarian function suppression. SD, stable disease. PD, progressive disease.

We obtained clinical characteristics and treatment history from six out of the eleven patients with reversion events with patients' consent (Figure 3). All of them received PARP inhibitor or platinum-therapy prior to reversion detection. Four patients (P2, P4, P6 and P10) acquired reversion mutations after the failure of the treatment with a PARP inhibitor. Two patients (P3 and P7) had somatic reversion mutations identified after progressing on platinum-based treatment, of whom P7 also showed primary resistance to the subsequent olaparib treatment. Our findings show that acquired reversion of HRR germline mutations indicated poor response to platinum-based therapy or PARP inhibition.

Patient 2 was diagnosed with metastatic breast cancer (luminal B) in June 2017 at the age of 42 years. She subsequently received 6 cycles of vinorelbine and capecitabine combination, followed by fulvestrant and ovarian function suppression (OFS), and achieved stable disease (SD). After progressive disease (PD) in August 2018, the second-line treatment shifted to the combination of liposomal doxorubicin, cyclophosphamide and bevacizumab. Subsequently, the patient received palliative surgery and was treated with olaparib-based regimens based on the germline mutation *BRCA1* p. L1252fs detected via NGS. After an SD lasting for 7 months, the disease progressed when a somatic reversion mutation *BRCA1* p. P1238fs was identified. Of note, in the course of treatment for metastatic breast cancer, the patient was also detected with non-metastatic ovarian cancer in Dec 2017. She underwent resection and received olaparib treatment following paclitaxel+carboplatin for ovarian lesion (Figure 3A).

Patient 3 with metastatic triple-negative breast cancer (TNBC) received four lines of platinum-based chemotherapy. NGS performed at PD revealed a germline mutation *PALB2* p. T827fs, accompanied by four confirmed somatic reversion mutations and 4 putative reversion mutations (Figure 3B).

Patient 4 was 36-year-old and diagnosed with stage IV breast cancer (luminal B) in February 2017. She received the treatment with tamoxifen, followed by the combination of fulvestrant, cyclophosphamide and epirubicin. Upon the disease progression in August 2018, she started receiving a PARP inhibitor (IMP4297) until January 2019. The germline mutation *BRCA2* p. C2817* and somatic reversion mutation p. C2817S were identified upon PD (Figure 3C).

Patient 6, 46-year-old, was detected with TNBC in September 2018. NGS result suggested the presence of a germline mutation *BRCA1* p. C1146fs. She received six cycles of carboplatin-based chemotherapy and achieved SD. Subsequently, olaparib was administrated as maintenance therapy and the disease remained stable until July 2019. Upon PD, the patient shifted to nab-paclitaxel and anti-PD-1 inhibitor. NGS was performed when the disease progressed again and indicated the emerging of somatic reversion mutation *BRCA 1* p. Q1144_P1150del (Figure 3D).

Patient 7, who was diagnosed with metastatic breast cancer (luminal B), received three lines of chemotherapy followed by endocrine therapy with goserelin and anastrozole. Subsequently the treatment was switched to fulvestrant and goserelin. In the meantime, the patient also underwent thoracic perfusion with endostatin and lobaplatin. After the disease progressed, she received palbociclib and exemestane until April 2019 when the treatment was switched to apatinib. Meanwhile, NGS was performed and showed concomitant germline mutation *BRCA2* p. Q2960* and somatic reversion mutation p. Q2960K. The patient then received olaparib treatment upon the progression of apatinib regimen but developed PD rapidly (Figure 3E).

Patient 10 was a 73-year-old male with metastatic pancreatic cancer. After surgery, the patient received treatment with gemcitabine and tegafur. After PD, he underwent two sequential transarterial chemoembolizations (TACEs) with gemcitabine+raltitrexed and gemcitabine +paclitaxel albumin, respectively. NGS screening indicated the presence of germline mutation of *BRCA2* p. Q1037* in February 2019 after the disease progressed. Subsequently, the patient received the olaparib and anlotinib combination following the olaparib single agent and achieved a partial response with a PFS of 12 months. Four somatic reversion mutations in *BRCA2* were identified upon the failure of olaparib regimen (Figure 3F).

## Discussion

We carried out a retrospective study investigating reversion mutations in 15 HRR genes in a large cohort of Chinese pan-cancer patients across multiple cancer types. 654 cases out of 23,375 (2.8%) pan-cancer patients were identified with HRR germline mutations. We observed reversion mutations emerging in 1.7% (11/654) of the patients with HRR germline mutations. Specifically, the prevalence of reversion was 8.0%, 6.2%, 3.2% and 0.3% in breast cancer, pancreatic cancer, ovarian cancer and lung cancer, respectively. On the other hand, 3.8%, 3.5% and 2.0% of *BRCA1*, *BRCA2* and *PALB2* germline mutation patients were identified with reversion events, respectively. Our study is a large assessment of reversion mutation prevalence of 15 HRR germline mutations in Chinese patients. It is also one of the few studies to assess reversion mutations across multiple tumor types.

Of note, the lower incidence of reversion mutation observed in our cohort is largely attributable to that we did not screen for patients with resistance to platinum-based or PARPi treatment, which will tremendously enrich the patients with reversion events. A recent study identified reversion mutations in 12 out of 1,308 unselected germline or somatic *BRCA1/2* mutant tumors from the MSKCC database 29, which is comparable with our study (1.7% vs 0.9%, P=0.118). Tobalina *et al.* reported a meta-analysis including 327 *BRCA1/BRCA2* mutant patients with ovarian cancer, breast cancer, pancreatic cancer or prostate cancer, who progressed on platinum-based or PARP inhibitor treatment. They observed an overall reversion incidence of 26% and the prevalence varied from 20%-40% across different cancers except for prostate cancer that showed a high prevalence of 81.8%. The prevalence of reversion events was 22.0% and 30.7% in *BRCA1* and *BRCA2* in their study, respectively 27. We failed to identify any reversion event in our prostate cancer cohort, probably due to the small number of patients detected with germline primary mutations (n=8).

Interestingly, we discovered one case (P11) out of the 313 HRR germline-mutant lung cancers harboring a reversion mutation *BRCA2* c.2978G>C p.W993S. Similarly, BRCA reversion mutations have recently been reported in two non-canonical BRCA-associated cancers: lung adenocarcinoma (n=1) and gastroesophageal junction adenocarcinoma (n=1), after the failure of platinum-based therapy 29. Particularly interesting is the two cases (P2 and P8) with multiple primary cancers, breast and ovary, out of the 11 patents with reversion mutations. Reversion mutations *BRCA1* c.3710_3711dup p.P1238fs and *BRCA1* c.67G>C p.E23Q were identified separately. It has been reported two primary cancers with breast-ovary is one of the most common (11.7%) cancer pairs in Chinese patients with multiple primary malignancies 30. For the breast cancer with *BRCA1* and *BRCA2* mutations, there is a strong association with an increased risk for a second breast or ovarian cancer 31.

Among the 25 reversion mutations we identified, 48% of the reversion mutations were pure deletions causing frameshift and in-frame mutations, followed by missense SNV (40%). This observation is in line with the finding that compared with missense and splice-site mutations, truncating pathogenic mutations in *BRCA1/2* are more prone to revert 11, 28. Similarly, Tobalina *et al.* showed that most revision events were deletions in both *BRCA1* (47/80) and *BRCA2* (170/219). SNV was the second common type of reversions in *BRCA1* (n=19), whereas insertion (n=18) and Indel (n=19) reversions occurred more frequently than SNV (n=8) in *BRCA2* 27. Moreover, the signature of MMEJ repair (microhomology length>1bp) was observed in 58.3% of reversion deletions in our study, compared with that of 30%-70% previously reported in *BRCA1/2* deletions 27, 28. Collectively, these observations underscore the essential role of the MMEJ-driven repair mechanism and suggest the involvement of other DNA repairing processes in generating reversions. Interestingly, we observed a positive correlation between the deletion length and the microhomology length (Figure 1C), which was not seen by Tobalina et al. 27. The discordance may be partially due to the fact that we only evaluated reversion deletions, most of which occurred in *PALB2*, while Tobalina *et al.* analyzed both primary and reversion deletions in *BRCA1/2.* Nevertheless, the limited number of reversions identified in our study attenuates the strength of our finding, making it necessary to further investigate this correlation with an expanded sample size.

All the six patients with reversion mutations, whose disease courses were available in our study, underwent the treatment with a platinum-based regimen and/or PARP inhibitor prior to the identification of somatic reversion mutation. It is usually presumed that the reversion mutation emerging after platinum-based or PARP inhibitor treatment is induced by the treatment exerted pressure 4, 5. Lin *et al.* also reported *BRCA* reversion mutations from pretreatment samples in 18% platinum-refractory and 13% platinum-resistant high-grade ovarian carcinomas, which also predicted an inferior PARP inhibitor rucaparib survival 11, reserving the plausibility that reversion mutations could emerge primarily. In our study, patient 7, whose reversion mutation was identified after thoracic perfusion with lobaplatin, showed resistance to the following olaparib treatment. Conceivably, monitoring HRR mutation status along the disease course could be beneficial especially for informing resistance mechanisms and guiding subsequent therapies 32.

Our study has several limitations. Despite a large cohort encompassing over 17 cancer types recruited in the study, patients with lung cancer constituted the majority, resulting in a limited number of patients for most cancer types. Therefore, the capability of detecting reversions has been largely attenuated in these cancers (prostate cancer etc.). Moreover, due to the retrospective nature of the study, most patients lacked paired baseline/pretreatment and post-progression samples for genomic profiling. Thus we were only able to identify reversion events emerging on germline primary mutations while reversion on somatic primary mutation was not investigated in this study. Our study lacks of functional assay to elucidate the mechanisms and the direct impact of reversion mutations on cancer cells.

In the future, well-designed prospective studies that longitudinally monitor patients' mutation profile along the disease course will better track the emergence of reversion events and improve our understanding of the underlying mechanism, thus providing insight into the potential management of drug resistance in HRR-deficient tumors.

## Conclusion

This retrospective study demonstrated that the reversion mutations were observed in three HRR-associated genes (*BRCA1, BRCA2* and *PALB2*) with four cancer types (breast cancer, pancreatic cancer, ovarian cancer, and lung cancer) from this Chinese pan-cancer patient cohort. The reversion mutations frequently occurred after resistance to platinum-based chemotherapy and/or PARP inhibitor, and may predict poor outcome from ensuing PARP inhibition therapy. Therefore, monitoring HRR mutation status along the course of the disease could be beneficial especially to informing resistance mechanism and guiding subsequent therapies.

## Supplementary Material

Supplementary figure and table.

## Figures and Tables

**Table 1:** Next-generation sequencing by cancer type and germline mutations frequency.

Cancer type	Patients analyzed	Patients with HRR germline mutations	Mutation frequency
Ovarian cancer	656	93	14.2%
Breast cancer	1024	100	9.8%
Endometrial adenocarcinoma	169	11	6.5%
Pancreatic cancer	269	16	5.9%
Prostate cancer	158	7	4.4%
Cervical cancer	189	8	4.2%
Malignant melanoma	74	3	4.1%
Cancer of biliary tract	201	8	4.0%
Liver cancer	197	7	3.6%
Colorectal cancer	1282	38	3.0%
Stomach/esophagus cancer	732	18	2.5%
Head-neck cancer	137	3	2.2%
Kidney cancer	153	3	2.0%
Lung cancer	17029	313	1.8%
Glioma	76	1	1.3%
Sarcoma	226	1	0.4%
Bladder cancer	84	0	0.0%
Others	809	31	3.8%
Total	23375*	654**	2.8%

* 90 patients with multiple primary cancers. ** 6 patients with two primary cancers: 3 cases of breast and ovarian dual cancers, 1 case of ovarian and cervical dual cancers, 1 case of ovarian and endometrial dual cancers, and 1 case of lung and cervical dual cancers.

**Table 2:** Different HRR reversion mutations across different cancer types.

	Total HRR genes			BRCA1			BRCA2			PALB2			Other HRR genes	
Cancer type	Patients with HRR germline mutations	Patients with reversions	Reversion frequency	Patients with HRR germline mutations	Patients with reversions	Reversion frequency	Patients with HRR germline mutations	Patients with reversions	Reversion frequency	Patients with HRR germline mutations	Patients with reversions	Reversion frequency	Patients with HRR germline mutations	Patients with reversions
Lung cancer	313	1	0.3%	28	0	0.0%	75	1	1.3%	22	0	0.0%	188	0
Breast cancer	100	8	8.0%	26	4	15.4%	39	3	7.7%	14	1	7.1%	23	0
Ovarian cancer	94	3	3.2%	59	3	5.1%	22	0	0.0%	0	0	NaN	13	0
Pancreatic cancer	16	1	6.2%	2	0	0.0%	8	1	12.5%	1	0	0.0%	5	0
Total patient	654*	11**	1.7%	130***	5**	3.8%	144	5	3.5%	49	1	2.0%	305****	0

* 6 patients with multiple primary cancers. ** 2 patients with breast and ovarian dual primary cancers, both harboured *BRCA1* reversion mutation. *** 4 patients with two primary cancers: 3 cases of breast and ovarian dual cancers, 1 case of ovarian and cervical dual cancers. **** 2 patients with two primary cancers: 1 case of ovarian and endometrial dual cancers, and 1 case of lung and cervical dual cancers.

**Table 3:** List of 11 cases identified with somatic reversion mutation(s).

Patient	Sex	Age	Cancer type	Clinical stage	Gene	Germline mutation	Somatic reversion mutations/status
P1	F	47	Breast	IV	*BRCA1*	c.2341G>T p.E781*	c.2307_2348del p.S770_I783del/confirmed c.2339_2347del p.Q780_I783delinsL/confirmed c.2343A>T p.E781Y/confirmedc.2342A>C p.E781S/confirmedc.2341G>C p.E781Q/confirmed
P2	F	42	Breast/ovarian	IV(Br.)/II(ov.)	*BRCA1*	c.3754_3755del p.L1252fs	c.3710_3711dup p.P1238fs/confirmed
P3	F	58	Breast	IV	*PALB2*	c.2480_2481del p.T827fs	c.2469_2492del p.C824_L831del/confirmedc.2466del p.Q822fs/confirmedc.2450_2465del p.T817fs/confirmedc.2457del p.E820fs/confirmedc.2339del p.G780fs/putativec.2322_2325del p.K774fs/putativec.2313del p.S771fs/putativec.2298_2301del p.C768fs/putative
P4	F	36	Breast	IV	*BRCA2*	c.8451T>A p.C2817*	c.8450G>C p.C2817S/confirmed
P5	F	39	Breast	III	*BRCA2*	c.2870del p.N957fs	c.2837_2838insG p.D946fs/confirmed
P6	F	46	Breast	IV	*BRCA1*	c.3436_3439del p.C1146fs	c.3430_3450del p.Q1144_P1150del/confirmed
P7	F	36	Breast	IV	*BRCA2*	c.8878C>T p.Q2960*	c.8878C>A p.Q2960K/confirmed
P8	F	53	Breast/ovarian	IV	*BRCA1*	c.66dup p.E23fs	c.67G>C p.E23Q/confirmed
P9	F	54	Ovarian	IV	*BRCA1*	c.4065_4068del p.N1355fs	c.4096G>A p.G1366S/confirmed
P10	M	73	Pancreatic	IV	*BRCA2*	c.3109C>T p.Q1037*	c.3109_3111del p.Q1037del/confirmedc.3109_3111delinsTAC p.Q1037Y/confirmedc.3109_3110delinsTC p.Q1037S/confirmedc.3109C>G p.Q1037E/confirmed
P11	F	63	Lung	IV	*BRCA2*	c.2979G>A p.W993*	c.2978G>C p.W993S/confirmed

## Abbreviations

HRR: homologous recombination repair
HRD: homologous recombination deficiency
WBC: white blood cell
P/LP: pathogenic/likely pathogenic
cfDNA: cell-free DNA
NGS: next-generation sequencing
MMEJ: microhomology-mediated end-joining
SNV: single nucleotide variant
