# Supplementary material for: Comprehensive Analysis of Somatic Reversion Mutations in Homologous Recombination Repair (HRR) Genes in A Large Cohort of Chinese Pan-cancer Patients
Source: J Cancer. 2022 Jan 9;13(4):1119–29. doi: 10.7150/jca.65650 (PMC8899361; doi:10.7150/jca.65650)
Supplement: Supplementary file 1 — Supplementary figure and table. [file jcav13p1119s1.pdf]

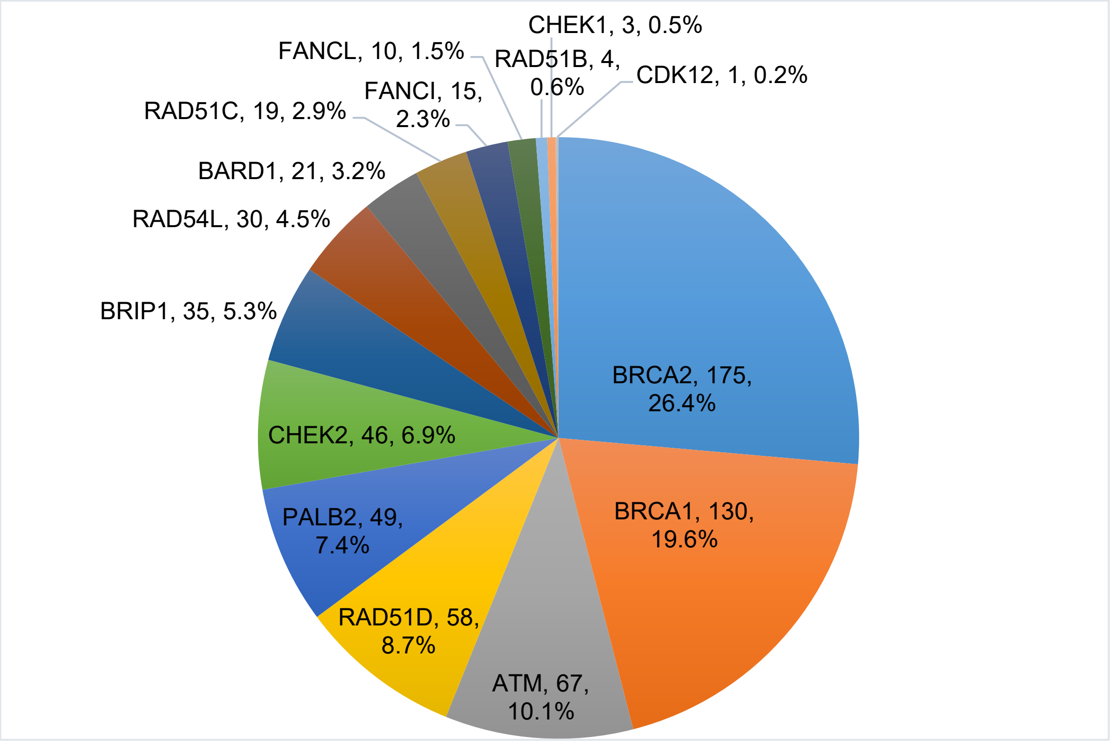

Supplementary Figure 1. Prevalence and distribution of P/LP germline mutations among 15 HRR genes.

Supplementary Table 1. Clinicopathologic characteristics of patients in the total cohort and some major types in this study.

	Overall (n = 23375)	Lung cancer (n = 17029)	Breast cancer (n = 1024)	Ovarian cancer (n = 656)	Pancreatic cancer (n = 269)	Patients with HRR germline mutations (n = 654)
**Gender**						
Female	10929 (46.6%)	7509 (44.1%)	997 (97.4%)	604 (92.1%)	108 (40.1%)	379 (58.0%)
Male	11490 (49.0%)	8905 (52.3%)	0 (0.0%)	0 (0.0%)	153 (56.9%)	246 (37.6%)
Unknown	1046 (4.5%)	615 (3.6%)	27 (2.6%)	52 (7.9%)	8 (3.0%)	29 (4.4%)
**Age**						
Age (Mean±SD	59.45±12.16	60.97±11.35	50.09±10.88	54.52±11.00	60.24±11.35	57.04±12.51
**Stage**						
I	1201 (5.1%)	903 (5.3%)	200 (19.5%)	13 (2.0%)	2 (0.7%)	34 (5.2%)
II	2175 (9.3%)	1438 (8.4%)	55 (5.4%)	118 (18.0%)	56 (20.8%)	61 (9.3%)
III	6848 (29.2%)	4790 (28.1%)	258 (25.2%)	286 (43.6%)	78 (29.0%)	210 (32.1%)
IV	12511 (53.3%)	9478 (55.7%)	464 (45.3%)	199 (30.3%)	132 (49.1%)	323 (49.4%)
Unknown	730 (3.1%)	420 (2.5%)	47 (4.6%)	40 (6.1%)	1 (0.4%)	26 (4.0%)
